# Psychological distress among early career university teachers: an ecological perspective

**DOI:** 10.3389/fpsyg.2026.1815223

**Published:** 2026-05-29

**Authors:** Maojun Duan

**Affiliations:** School of Foreign Languages, Sichuan Normal University, Chengdu, China

**Keywords:** Bronfenbrenner’s ecological systems theory, early career university teachers, educational psychology, psychological distress, qualitative case study

## Abstract

Under China’s “Double First-Class” initiative, higher education in mainland China has become increasingly performance-driven, competitive, and metric-oriented. In this context, early career university teachers experience psychological distress not merely as an individual problem, but as a systemic outcome shaped by relational, institutional, cultural, and temporal pressures. Drawing on Bronfenbrenner’s ecological systems theory, this qualitative case study examines how factors across the micro-, meso-, exo-, macro-, and chrono-systems jointly shape the psychological distress of early career university teachers in Sichuan Province, mainland China. Data were collected through in-depth semi-structured interviews with 12 PhD-holding early career university teachers within their first 3–5 years of appointment. Interview data were analyzed using reflexive thematic analysis to identify patterns of meaning across ecological layers. The findings reveal a cascading pattern of distress, characterized by emotional exhaustion in strained interpersonal relationships, role confusion arising from overlapping professional demands and work-life imbalance, value conflicts under managerialist governance, quantification pressure driven by utilitarian evaluation standards and *neijuan* culture, and age anxiety as a temporal amplifier of cumulative stress. This study contributes to educational psychology by showing how early career teachers’ distress is produced through interlocking ecological pressures and may become consequential for instructional practices.

## 1 Introduction

In contemporary higher education, psychological distress among university teachers has become an increasingly important issue (Mukhtar, 2024; Akinlotan et al., 2025). As universities are increasingly shaped by global ranking competition, performance accountability, and metric-oriented governance, academic work has become more intensive, uncertain, and emotionally demanding (Guthrie et al., 2024). These changing work conditions may affect the psychological conditions under which teaching is planned, interpreted, and enacted (Lazarides et al., 2025). From an educational psychology perspective, psychological distress should not be understood merely as a matter of teachers’ mental health or individual wellbeing, but also as a condition that may shape the cognitive, motivational, and belief-related processes underlying instructional practices (Walker and Debus, 2002; Borg, 2003). In this sense, distress may influence how university teachers perceive classroom demands, sustain pedagogical engagement, and make sense of worthwhile teaching, particularly when work-related stressors undermine teaching engagement (Xu et al., 2023).

This issue is especially salient for early career university teachers. During the transition from doctoral training to full-time academic work, early career teachers are expected to establish research profiles, develop teaching competence, obtain grants and publications, participate in administrative service, and adapt to institutional evaluation systems within a relatively short period (Stratford et al., 2024; Marck et al., 2024). In mainland China, these pressures have been further intensified under the “Double First-Class”^1^ initiative and performance-oriented reforms, which have increased expectations for rapid research productivity, competitive advancement, and measurable academic output (Zheng and Li, 2025). At the same time, early career university teachers continue to shoulder substantial teaching and administrative responsibilities. The growing use of probationary assessment and “up-or-out”^ 2^ evaluation systems has made their early professional stage especially uncertain and high-stakes (Yang et al., 2024). Therefore, Chinese higher education provides a particularly informative and dynamic context for examining how psychological distress is shaped through the interaction of institutional evaluation, academic competition, teaching responsibilities, and career uncertainty.

Although substantial research has explored university teachers’ stress and wellbeing, much of the existing literature has focused on discrete workplace stressors, such as workload intensification, job insecurity, publication pressure, evaluative stress, and work-family conflict, all of which are associated with anxiety, burnout, and emotional exhaustion (Wang et al., 2025). Some studies have examined specific relationships among workplace variables, such as how perceptions of organizational politics influence job anxiety (Haider et al., 2020), or how student-teacher conflict contributes to emotional exhaustion (Olivier et al., 2024). However, these studies tend to treat stressors as relatively isolated variables. They offer limited qualitative insight into how relational, institutional, sociocultural, and temporal pressures interact across ecological levels to shape the psychological distress of early career university teachers in mainland China.

Recent studies have begun to adopt ecological or socio-ecological psychology perspectives to understand teachers’ emotions, wellbeing, and professional challenges (Zhang and Yusof, 2024; Ghasemi, 2025; Irgin, 2025). These studies suggest that teachers’ psychological experiences are not produced by individual coping alone but are embedded in broader environmental systems. Nevertheless, qualitative research explicitly applying Bronfenbrenner’s ecological systems theory to psychological distress among early career university teachers remains limited. In particular, existing literature has not sufficiently explained how interpersonal strain, role conflict, managerialist governance, performance-oriented culture, and age-related temporal pressure become linked across levels and translated into sustained psychological distress. Nor has it fully clarified how such distress may reshape teachers’ instructional practices from an educational psychology perspective.

To address this gap, the present study draws on Bronfenbrenner’s ecological systems theory as a contextual framework for examining psychological distress as an ecologically embedded phenomenon (Bronfenbrenner, 1979). Rather than treating distress as a set of isolated workplace stressors, the study investigates how pressures across the micro-, meso-, exo-, macro-, and chrono-systems interact to produce psychological distress among early career university teachers. In doing so, it links ecological conditions in higher education to the educational-psychological mechanisms through which instructional practices may be influenced, particularly teacher cognition, teacher motivation, and beliefs about teaching.

## 2 Literature review

### 2.1 Defining psychological distress

Psychological distress generally refers to adverse emotional states that interfere with individuals’ daily functioning without necessarily meeting the diagnostic criteria for mental disorders. From a psychological perspective, it encompasses subjective experiences such as anxiety, frustration, depression, and emotional exhaustion, and often emerges when individuals perceive that environmental demands exceed their available coping resources (Kessler et al., 2002; Viertiö et al., 2021). In this sense, psychological distress captures how strain is felt and experienced at the individual level.

From a sociological perspective, however, psychological distress is not understood solely as an internal emotional condition. Rather, it is shaped by socially patterned stressors embedded in everyday life, including role strain, limited social support, institutional arrangements, and broader cultural expectations (Thoits, 2010). Pearlin’s (1989) stress process framework conceptualizes psychological distress as the outcome of sustained exposure to stressors that are socially distributed and mediated by coping resources and support systems. Similarly, Mirowsky and Ross (2003) emphasize that psychological distress is closely linked to life conditions and social structures rather than to individual weakness alone. This perspective shifts attention from distress as a purely personal problem to distress as a response to structurally organized pressures.

Taken together, psychological distress can be understood as both a psychological experience and a socially embedded condition. It is psychological because it is lived by individuals as anxiety, frustration, and emotional exhaustion. It is sociological because these experiences are shaped by the institutional, relational, and cultural contexts in which individuals work and live. For early career university teachers, this integrated understanding is especially important because their psychological distress extends beyond personal coping and is closely tied to performance evaluation, role conflict, organizational expectations, and broader sociocultural pressures. This combined perspective therefore provides an appropriate conceptual foundation for examining psychological distress as an ecologically embedded phenomenon in this study.

### 2.2 Linking psychological distress to ecological stress among early career university teachers

Psychological distress among early career university teachers should not be understood simply as an isolated psychological problem. Rather, existing research suggests that it is closely connected to ecological stress embedded in the wider environments in which academic work is carried out. Studies of mental health in academia have shown that anxiety, emotional exhaustion, reduced wellbeing, and disengagement are often shaped by the broader conditions of academic life rather than by individual coping alone (Nicholls et al., 2022). Research on teacher wellbeing has likewise emphasized that teachers’ psychological experiences are situated within contextual conditions, including interpersonal relations, organizational arrangements, and everyday work environments (Hascher and Waber, 2021). From this perspective, psychological distress can be understood as the psychological manifestation of ecological stress, that is, of pressures arising from the interconnected settings in which early career university teachers work and live. This understanding is also consistent with the current argument of this study that workplace stress should be seen as part of a wider stress ecology rather than as a separate phenomenon.

Existing studies further suggest that this ecological stress operates across multiple levels. At the interpersonal level, limited collegial support, weak mentoring, and strained workplace relationships may undermine wellbeing, especially for academics at the beginning of their careers (Stratford et al., 2024). At the organizational level, heavy workload, teaching-research tension, limited institutional support, work-life conflict, and job insecurity have been repeatedly identified as important sources of strain in higher education (Kinman and Jones, 2008). For early career university teachers, these pressures are intensified by tenure-track and “up-or-out” arrangements, which compress evaluation periods and heighten publication pressure and career uncertainty (Yang et al., 2024). At the sociocultural level, performance-oriented reforms, metric-based evaluation, and competitive academic norms have become increasingly consequential. Li and Kou (2018) report that psychological stress among university teachers in China is closely associated with work-related pressures, while Xu et al. (2023) show that challenge-hindrance stressors are significantly related to job satisfaction and teaching engagement in Chinese universities. Qualitative research on the “Double First-Class” initiative further indicates that intensified competition and *neijuan* culture have become important features of academic life and wellbeing in Chinese universities (Zhang et al., 2024). Finally, these pressures are also temporal. Early career university teachers often work under compressed probationary timelines, continuing assessment, and uncertain career futures, so stress is experienced not only in the present but also as anticipation of future judgment and possible exclusion. In this respect, early career vulnerability is shaped jointly by workload, career uncertainty, and organizational culture across time (Marck et al., 2024). Taken together, these studies suggest that workload, job insecurity, publication pressure, work-family conflict, performance reform, *neijuan* culture, and age-related deadlines should not be treated as disconnected stressors, but as interrelated forms of ecological stress that may culminate in psychological distress.

Despite this growing body of literature, an important gap remains. Existing studies have identified many correlates of distress, but they have more often treated these as relatively discrete workplace variables than examined how stressors across interpersonal, organizational, sociocultural, and temporal levels interact with one another. Although recent studies have begun to adopt ecological perspectives to examine teachers’ emotional experiences and wellbeing, qualitative research explicitly explaining how multiple ecological pressures jointly shape the psychological distress of early career university teachers remains limited. Likewise, current literature has not fully explained how relational strain, institutional governance, performance-oriented reform, and temporal pressure become linked across levels and translated into sustained psychological distress, nor how such distress may become consequential for teachers’ instructional practices. This gap points to the need for a clearer theoretical framework that can explain the stressors early career university teachers face and the ways these stressors become interconnected within a broader ecology. It is for this reason that the present study turns to Bronfenbrenner’s ecological systems theory in the following section.

### 2.3 Understanding psychological distress from Bronfenbrenner’s ecological systems theory perspective

Understanding psychological distress among early career university teachers requires a framework capable of capturing the complex, layered environments in which their work is situated. Bronfenbrenner’s ecological systems theory provides such a lens by conceptualizing human development as embedded within multiple, interrelated environmental systems, including the micro-, meso-, exo-, macro-, and chrono-systems (Bronfenbrenner, 1979). In the present study, this ecological perspective is used to examine how ecological factors across systems interact to shape psychological distress, and how such distress is linked to possible changes in instructional practices through educational-psychological mechanisms.

Existing research provides support for this perspective. Teacher wellbeing has increasingly been shown to be shaped by contextual factors such as interpersonal relationships, organizational climate, workload, and institutional support, rather than by individual coping alone (Hascher and Waber, 2021). Studies on early career academics likewise suggest that work overload, career uncertainty, mentoring, organizational culture, and work-life tensions jointly influence wellbeing and professional flourishing (Marck et al., 2024; Stratford et al., 2024). More directly, recent ecological studies have shown that teachers’ emotional experiences and occupational challenges can be understood across multiple environmental systems (Ghasemi, 2025; Irgin, 2025). This perspective has also been applied to novice university teachers in China, whose emotional experiences have been found to be shaped across the ecological systems (Zhang and Yusof, 2024). Although these studies do not always focus specifically on psychological distress, they collectively show that teachers’ psychological experiences are shaped by interrelated ecological conditions rather than by individual factors alone. However, some gaps remain. Ecological or socio-ecological perspectives have more often been used to examine teacher emotions, wellbeing, and professional development than psychological distress as a distinct, non-diagnostic, and contextually embedded phenomenon. Qualitative studies explicitly applying Bronfenbrenner’s ecological systems theory to psychological distress among early career university teachers in higher education remain limited. This gap is especially important in mainland China, where interpersonal demands, institutional evaluation systems, policy reforms, and culturally embedded career expectations intersect in ways that may intensify psychological distress.

Within Bronfenbrenner’s framework, the micro-system refers to the most immediate environments in which individuals engage in face-to-face interactions, encompassing “activities, roles, and interpersonal relations experienced by the developing person in a given setting” (Bronfenbrenner, 1979, p. 22). For early career university teachers, the micro-system includes everyday teaching practices, interactions with students, colleagues and leaders. These experiences constitute the most direct sources of emotional strain, particularly when teaching demands or collegial relationships are experienced as challenging or unsupportive.

The meso-system comprises “the interrelations among two or more settings in which the developing person actively participates” (Bronfenbrenner, 1979, p. 25). For early career university teachers, the meso-system involves the connections between different professional domains, such as the relations among teaching responsibilities, research expectations, administrative duties, and personal life. Confusion between these domains—such as conflicting expectations for teaching quality and pressures to publish—can heighten psychological distress by exacerbating role overload.

The exo-system includes social structures and institutional arrangements that do not directly involve the individual but nonetheless exert indirect influence on their experiences (Bronfenbrenner, 1979, p. 25). For early career university teachers, the exo-system includes institutional evaluation policies, tenure-track systems, workload arrangements and resource allocation systems. Although early career university teachers may have limited control over these conditions, such policies can profoundly shape their job security, career prospects, and psychological wellbeing.

The macro-system refers to the overarching cultural, ideological, and policy frameworks that shape social institutions and practices (Bronfenbrenner, 1979, p. 81). For early career university teachers, the macro-system encompasses broader sociocultural values and discourses surrounding academic excellence, meritocracy, productivity, and competition. In many higher education systems, these values are reinforced through national policy agendas and global ranking regimes, creating normative expectations that may legitimize excessive workloads and intensify psychological distress.

The chrono-system captures the temporal dimension of ecological systems, emphasizing how individuals’ experiences are shaped by changes and transitions over time (Bronfenbrenner, 2005, p. 83). For early career university teachers, psychological distress may evolve across different career stages, shaped by age-related anxiety and cumulative exposure to evaluation cycles. The chrono-system highlights that distress is not static but develops through the interaction of past experiences, present conditions, and anticipated future trajectories.

Accordingly, through the lens of ecological systems theory, the present study seeks to examine how psychological distress among early career university teachers is shaped across multiple, interrelated systems within their professional environments. By adopting this framework, the study aims primarily to identify the ecological conditions under which psychological distress emerges. It also provides a basis for discussing the possible implications of such distress for instructional practices.

## 3 Methodology

### 3.1 Research design

This study adopts a qualitative case study design (Creswell and Poth, 2018), theoretically grounded in Bronfenbrenner’s ecological systems theory (Bronfenbrenner, 1979). It conceptualizes psychological distress as a multi-level, contextually embedded phenomenon and examines it within the bounded case of early career university teachers working in mainland Chinese universities in Sichuan. Given the complexity, contextual embeddedness, and processual nature of psychological distress, a qualitative case study design is particularly appropriate, as it enables in-depth exploration of participants’ lived experiences and facilitates an understanding of how distress is constructed within real-life contexts over time (Merriam and Tisdell, 2016; Yin, 2018). The study is guided by two questions: What ecological factors across the micro-, meso-, exo-, macro-, and chrono-systems contribute to the psychological distress of early career university teachers in mainland China? How do these ecological factors interact across systems and become educationally consequential through teachers’ cognition, motivation, and beliefs about teaching?

### 3.2 Participants and sampling

Purposive sampling was employed to recruit information-rich participants directly relevant to the research focus (Patton, 2015). Participants were identified through two main channels: a provincial teacher training program in Sichuan in which both the researcher and prospective participants had taken part, and professional connections developed through academic networks. Invitations were sent by email or direct message. To be included, participants had to (1) hold a full-time university teaching or research position at a university in Sichuan Province, mainland China, (2) have completed a PhD, (3) be within 3–5 years of appointment and (4) be subject to formal evaluation, contract renewal, or probationary assessment. Sampling also sought variation across discipline, gender, and appointment type to capture diverse manifestations of psychological distress under different institutional conditions. Twelve teachers met these criteria and agreed to participate. Recruitment continued until substantial repetition in the interview data suggested sufficient depth for this qualitative case study. In this study, “early career” was operationalized as being within the first 3–5 years of a full-time university appointment in mainland China, which broadly corresponds to the probationary or tenure-track period in many Chinese universities. Participant details are presented in Table 1 using anonymized codes (P1–P12). To ensure ethical rigor, informed consent was obtained from all participants, pseudonyms were used, and identifying details were removed in accordance with established qualitative research ethics (Creswell and Poth, 2018).

**TABLE 1:** Profile of the participants.

No.	Gender	Age	Discipline	Academic rank	Appointment type	Description of psychological distress
P1	Male	32	Social sciences	Lecturer	Tenure-track (up-or-out)	High anxiety, sleep disturbance
P2	Female	35	Humanities	Associate professor	State-funded	Moderate anxiety, role conflict
P3	Male	33	Engineering	Lecturer	Contract-based	Extreme fatigue, intense research pressure
P4	Female	31	Astronomy	Assistant researcher	Tenure-track (up-or-out)	Depressive tendency, social withdrawal
P5	Male	35	Medicine	Lecturer	Tenure-track (up-or-out)	Long-term overwork, physical and mental exhaustion
P6	Female	30	Arts	Lecturer	State-funded	Marginalization, low professional identity
P7	Male	32	Economics	Lecturer	Tenure-track (up-or-out)	Severe insecurity, emotional instability
P8	Female	34	Literature	Lecturer	Tenure-track (up-or-out)	Strong feelings of guilt (work-family imbalance)
P9	Male	32	Education	Lecturer	State-funded	Frustration, perceived institutional injustice
P10	Female	33	Foreign languages	Lecturer	State-funded	Anxiety (teaching-research conflict)
P11	Female	29	Architecture	Lecturer	Tenure-track (up-or-out)	Career uncertainty, stress-related weight gain
P12	Female	33	Agriculture	Assistant researcher	Contract-based	Loneliness, intense peer competition

### 3.3 Data collection

Data were collected primarily through semi-structured interviews, which were well suited to exploring participants’ lived experiences and meaning-making processes within complex institutional contexts (Kvale and Brinkmann, 2009). Guided by Bronfenbrenner’s ecological systems theory (Bronfenbrenner, 1979), the interview protocol was designed to elicit accounts of psychological distress at multiple ecological levels, including, but not limited to, everyday teaching practices, collegial relationships, institutional evaluation systems and broader sociocultural expectations. The semi-structured interview protocol is presented in Appendix Table 1.

Twelve early career university teachers participated in one-to-one interviews lasting between 60 and 90 min. Interviews were conducted in Chinese, audio-recorded with participants’ consent, and transcribed verbatim. Open-ended questions encouraged participants to reflect on their teaching work, research demands, career trajectories, and emotional experiences, while follow-up prompts were used to clarify contextual details and temporal processes. To maintain linguistic equivalence, the transcripts and selected quotes were translated into English and cross-checked by two bilingual researchers. To enhance contextual understanding, interview data were complemented by analytic memos written during and after data collection. Data collection continued until sufficient depth and richness had been achieved to support the development of the analytic themes, indicated by the recurrence of patterns across participants. All procedures strictly adhered to established ethical guidelines for qualitative research.

### 3.4 Data analysis

Data were analyzed using reflexive thematic analysis, following the six-phase approach outlined by Braun and Clarke (2019). This method was selected for its flexibility and its suitability for interpreting patterns of shared meaning in participants’ accounts of psychological distress. Analysis began with repeated reading of the interview transcripts to develop familiarity with the data, during which the researcher recorded initial analytic observations and reflexive notes.

Initial codes were generated inductively from the interview transcripts to capture participants’ descriptions of emotionally significant experiences, institutional pressures, and temporal concerns. The analysis followed a theory-informed reflexive thematic analysis approach. Initial coding was conducted inductively to capture participants’ lived experiences of psychological distress without imposing predefined categories. In subsequent stages, these codes were iteratively interpreted and organized in relation to Bronfenbrenner’s ecological systems theory, allowing the mapping of emergent themes onto the micro-, meso-, exo-, macro-, and chrono-systems. In this sense, the ecological framework was used as an interpretive lens rather than a priori coding structure, enabling a balance between data-driven analysis and theoretically informed interpretation. Through this recursive process, codes were reviewed, combined, re-specified, and developed into broader themes that captured patterned meanings across participants’ accounts. Illustrative examples of this analytic process are presented in Table 2.

**TABLE 2:** Coding samples.

Original excerpt	Initial code	Developed theme	System level
“I spend a lot of time preparing for my classes, but my students are absorbed in their phones.”	Student disengagement in class	Emotional exhaustion in teaching interactions	Micro-system
“I feel like I can’t separate my work from my life at all, and there’s always an endless list of things to do every day.”	Blurred work-life boundary	Role confusion across work-life domains	Meso-system
“You don’t argue with the management. You adjust yourself, even if it means giving up what you believe good teaching or research should be.”	Institutional governance regime	Value conflicts in the logic of managerialism	Exo-system
“Everyone talks about excellence and productivity, but it feels like we’re just competing endlessly for numbers.”	Competition for numbers	Performance-oriented academic culture	Macro-system
“If I fail to secure a national-level grant before the age of 35, people assume that my academic future is over.”	Age-related career deadline	Temporal pressure and anticipatory career anxiety	Chrono-system

Throughout the analysis, ongoing critical reflection was central to the analytic process. The researcher critically examined analytic decisions, theoretical assumptions, and positionality through memo writing and repeated return to the data. Consistent with a reflexive thematic analysis approach, analytic rigor was pursued not through inter-coder reliability, but through reflexive memo writing, iterative engagement with the data, transparency in theme development, and careful alignment between participants’ accounts, emerging interpretations, and the ecological framework. Ethical considerations were integrated throughout the analytic process, with participants’ confidentiality preserved and all data handled in accordance with established qualitative research ethics.

## 4 Findings and discussion

### 4.1 Micro-system: emotional exhaustion in interpersonal relationships

At the micro-system level, psychological distress was most immediately experienced through emotionally demanding face-to-face relationships embedded in everyday life, particularly interactions with students, colleagues, and institutional leaders. These relationships required sustained emotional regulation, attentiveness, and professional self-management, yet were often experienced as one-sided, weakly supportive, or shaped by evaluative vulnerability. Under such conditions, routine interpersonal encounters gradually became a proximal mechanism through which high emotional investment, limited recognition, and restricted opportunities for recovery were translated into emotional exhaustion.

In teaching contexts, participants frequently reported a growing sense of frustration and diminished professional accomplishment. A recurring concern was students’ low levels of observable engagement during lectures, particularly the widespread use of smartphones, which participants experienced as undermining the reciprocal nature of teaching. As one participant noted, “I spend a lot of time preparing for my classes, but my students are absorbed in their phones. Sometimes I feel like I’m talking to myself” (P10). These experiences echo prior research, which indicates that perceived student disengagement is a key predictor of teachers’ emotional exhaustion (Hakanen et al., 2006), and that digital distractions can negatively affect both learning outcomes and instructors’ instructional confidence (Kuznekoff and Titsworth, 2013). Over time, such interactions eroded participants’ sense of teaching efficacy, and contributed to cumulative emotional exhaustion. Moreover, emotional exhaustion in teaching was not solely attributed to student behavior. Participants consistently described anxiety surrounding student evaluations of teaching, which fundamentally shaped how they enacted classroom authority. Several early career university teachers reported hesitating to intervene in disruptive situations due to concerns that negative feedback could adversely affect annual evaluations, promotion prospects, or contract renewal. One teacher explained, “Even when students disturb the class, I hesitate to intervene. If they’re unhappy, it will show up in the teaching evaluations” (P4). This dynamic reflects a broader paradox in higher education whereby evaluative mechanisms intended to enhance teaching quality instead intensify emotional labor and encourage risk-avoidant pedagogical practices (Hornstein, 2017). However, prior empirical research also suggests that student evaluations may function differently depending on how they are institutionally used. When employed formatively to support teaching improvement, they can serve as pedagogical resources rather than solely as stressors (Stein et al., 2021; Roxå et al., 2022). Consequently, emotional exhaustion emerged not simply from teaching demands, but from the sustained self-regulation required to balance pedagogical responsibility with ongoing evaluative vulnerability.

Beyond the teaching contexts, participants described a noticeable weakening of collegial relationships within their departments. Intensified research expectations and performance-oriented competition were perceived as fostering individualized work patterns and constraining opportunities for informal emotional support. Several participants noted that colleagues were preoccupied with meeting publication and research funding targets, leaving little space for shared wellbeing. As one participant observed, “Everyone is focused on their own papers and grants. We rarely talk about how we’re doing” (P12). This erosion of collegial support aligns with evidence that performance-oriented academic cultures can undermine social support networks that might otherwise buffer stress and emotional exhaustion, particularly for early career staff (Kinman et al., 2011; Wray and Kinman, 2021). The resulting sense of relational isolation further intensified emotional exhaustion within the micro-system. At the same time, prior research suggests that collegial interaction can serve as an important source of emotional and professional support when departmental climates are more collaborative, as shown in studies of early career academics’ flourishing and organizational support in higher education (Stratford et al., 2024).

Leadership expectations for early career university teachers are also a key factor contributing to emotional exhaustion. Participants highlighted the emotional burden arising from interactions with leaders regarding research productivity. Research expectations were described not as periodic goals but as permanently monitored requirements embedded in annual reviews, performance indicators, and informal reminders. Several participants noted that institutional leadership increasingly placed important research tasks—such as applications for national-level grants—on early career university teachers, positioning them as key contributors to departmental research performance. While often framed as opportunities for early career development, these expectations intensified pressure and heightened perceptions of accountability. As one participant explained, “We are required to submit annual applications to the National Social Science Fund of China. I’m always working—during evenings, weekends, and holidays” (P9). Such interactions rendered research work highly visible, thereby encouraging long working hours and fostering chronic anticipatory anxiety. These experiences show how broader evaluative pressures were transmitted through everyday interactions with leaders, turning routine communication about research into a proximal source of emotional strain. This pattern is consistent with research showing that managerial and performance-oriented accountability can intensify the visibility of academic work and heighten emotional pressure (Deem et al., 2007; Gill, 2016).

Overall, psychological distress often arose when interpersonal interactions in the micro-system felt one-sided—high effort with little feedback, recognition, or support. Participants invested substantial emotional, cognitive, and temporal resources, yet perceived limited opportunities for recovery, recognition, or relational support in return. In this sense, the micro-system functioned as a critical conduit through which strained relationships were translated into everyday emotional exhaustion, suggesting that psychological distress was closely tied to micro-level interpersonal strains rather than to individual shortcomings alone. These micro-level experiences also reshaped teacher cognition in classroom settings to some extent. Participants’ accounts suggest that strained interpersonal interactions can alter how teachers perceive and interpret classroom demands, weaken instructional self-efficacy, and increase defensive emotion regulation, thereby reducing confidence in classroom intervention and pedagogical responsiveness. These micro-level strains did not remain confined to everyday interactions; rather, as emotional resources were depleted, they spilled over into the meso-system, where competing role demands became harder to manage.

### 4.2 Meso-system: role confusion across multiple tasks

At the meso-system level, psychological distress was amplified by the intersection of teaching, research, administrative work, and family life. Rather than being experienced as separate domains, these settings overlapped in ways that intensified time scarcity, blurred work-life boundaries, and made competing expectations difficult to reconcile. As demands accumulated across contexts, participants were repeatedly forced to shift between incompatible roles, turning ordinary workload pressure into persistent role confusion and a chronic sense of inadequacy.

A defining feature of this meso-system was the intensification of administrative tasks, through which early career university teachers were repeatedly mobilized to absorb “miscellaneous” departmental tasks—supervising military training, drafting documents, organizing meetings, and preparing for inspections. Participants noted that this work was seldom formalized in workload models, yet it consumed substantial time and attention. As one interviewee observed, “Whenever there is something extra to be done, it usually goes to young teachers. Senior teachers are rarely required” (P11). Beyond these duties, load accumulation was further amplified by extensive administrative and service obligations—advising students, supervising internships, participating in teaching competitions, attending mandatory trainings, and completing institutionally assigned tasks—forms of essential but undervalued labor akin to “academic housework” that is disproportionately allocated to early-career teachers (Heijstra et al., 2017). Crucially, participants described this burden as unevenly distributed and difficult to avoid, so that administrative tasks routinely spilled over into time needed for teaching, research, and family responsibilities: “Senior colleagues can refuse. We can’t. Saying no means being labeled uncooperative” (P7). Together, these administratively mediated demands intensified time scarcity and deepened role conflict across teaching, research, and service, thereby escalating psychological distress.

Importantly, one participant framed overload as a structural positioning problem rather than a simple matter of being “busy.” They were simultaneously expected to meet key research indicators, maintain teaching competence, and remain compliant administrative actors. The participant framed this as a destabilizing identity question—“Am I a teacher, a researcher, or an administrator?” (P3)—because the institution’s signals pulled them in different directions, leaving them in a state of role confusion. Within China’s competitive higher education environment, intensified performance regimes magnified the perceived consequences of falling behind, making overload psychologically threatening rather than merely burdensome (Yang et al., 2024). Participants described a persistent anxiety that fulfilling one role adequately would inevitably undermine another, leaving them with a chronic sense of inadequacy and precariousness.

Meso-system tensions were also heightened by work-family role conflict, particularly during life stages involving marriage and early childrearing. Many participants expected greater temporal stability after completing doctoral studies, yet encountered the opposite as publication targets, evaluations, and funding applications continued to dominate daily life. One participant reflected that despite having a 3-year-old child, “Most of my time is still taken up by research deadlines and evaluations. I am physically present at home, but often absent. There is no boundary between work and family life” (P8). These pressures were especially acute in early childhood, when parental time and emotional investment are high (Craig and Powell, 2011), and were experienced as chronic in environments of continuous monitoring (Kinman and Jones, 2008). Female teachers further described intensified guilt and exhaustion linked to gendered caregiving expectations and the motherhood penalty in academia (Budig et al., 2012; Ward and Wolf-Wendel, 2012).

Taken together, these findings show that psychological distress at the meso-system level emerges from sustained negotiations of incompatible role expectations across multiple tasks, with limited structural support for reconciliation; the meso-system thus becomes a key site where institutional contradictions are lived and translated into enduring psychological distress. This meso-system pattern is educationally consequential because sustained role conflict may fragment teachers’ attentional resources and, more importantly, weaken teacher motivation for pedagogical work. When teaching, research, administrative work, and family responsibilities become difficult to reconcile, pedagogical engagement may increasingly give way to externally regulated task completion, leaving less time, energy, and commitment for lesson planning and student support. In this sense, meso-system role confusion functioned as a transmission point through which exo-system governance arrangements and macro-system performance norms were translated into lived distress in everyday life.

### 4.3 Exo-system: value conflicts in the logic of managerialism

At the exo-system level, psychological distress was shaped by university evaluation practice, governance regimes, and resource allocation systems that also redefined the psychological conditions under which teaching was understood and enacted. These institutional arrangements did not merely intensify workload or constrain professional autonomy; they also influenced how early career university teachers allocated attention, sustained motivation for pedagogical work, and interpreted the relative value of teaching within academic life.

A central tension arose from the gap between institutional discourse and evaluative practice. Although universities publicly emphasized teaching quality and student development, participants consistently reported that assessment systems rewarded research performance far more strongly than pedagogical commitment. This discrepancy created value conflict and gradually changed how early career teachers interpreted instructional practices. When teaching was symbolically affirmed yet practically marginalized, participants were pushed to view pedagogical effort as institutionally secondary, even when they personally valued instructional care. As one participant noted, “Teaching takes a lot of time, but it doesn’t really count. If I don’t publish, everything else becomes meaningless” (P3). This mattered for teaching because it could weaken the motivational basis for sustained pedagogical engagement.

These governance regimes created a widening gap between academic expectations and actual working conditions. While the university upheld high academic expectations, participants described daily workloads dominated by non-academic administrative demands that crowded out time for research development. In practice, administrative tasks eroded the time needed for research productivity, intensifying the sense of being stretched across irreconcilable expectations. For early career university teachers, this imbalance was not simply inefficient but demoralizing, especially given their limited capacity to refuse. One participant stated, “Honestly, I don’t want to do these tasks, but as an early career teacher, I have no choice but to comply” (P6). The resulting sense of constrained agency reflects the way bureaucratic rationality can displace professional judgment and autonomy (Weber, 1978). As professional autonomy narrowed, value conflict extended beyond the familiar tension between teaching and research to include a further tension between academic work and administrative compliance. This became an exo-systemic mechanism through which managerialism was translated into psychological distress. Nevertheless, empirical research indicates that accountability and performance-oriented arrangements may have differentiated effects. In some settings, clearer evaluation criteria and stronger research incentives have been associated with innovative work behavior and improved research performance (Yang et al., 2025). In the present study, however, participants more often encountered these arrangements as sources of constrained agency, because their implementation was experienced as rigid, unequal, and weakly supportive.

Exo-system pressures were compounded by perceived inequities in resource allocation systems and career advancement. Participants frequently described funding access, promotion opportunities, and academic titles as disproportionately favoring senior teachers, with some schemes formally requiring applicants to hold higher ranks. As one participant observed, “Many funding projects are only open to professors or associate professors… yet without publications and projects, it is impossible to gain promotion” (P9). This circular structure intensified feelings of injustice and powerlessness, reinforcing bureaucratic control over professional advancement (Abbott, 1988).

Psychologically, these arrangements fostered value conflict. Participants reported that professional value was increasingly reduced to measurable indicators (e.g., publication counts, grant income, assessment scores), while teaching quality, mentorship, and administrative contribution were symbolically acknowledged but practically marginalized. As one participant reflected, “You don’t argue with the management. You adjust yourself, even if it means giving up what you believe good teaching or research should be” (P9). The exo-system thus operated as a translation layer that converted abstract governance logics into concrete constraints, eroding professional discretion and intensifying distress already produced at the micro- and meso-system levels, while also linking closely to macro-system ideologies (Ball, 2012; Giroux, 2014). Moreover, when institutional reward systems repeatedly privilege measurable research outputs over pedagogical commitment, early career teachers may come to view teaching as symbolically valued but practically marginal, which may in turn weaken the motivational basis for sustained pedagogical investment. Thus, the exo-system functioned as a mediating layer through which macro-level policy and cultural logics were translated into the role confusion and emotional exhaustion experienced at the meso- and micro-system levels.

### 4.4 Macro-system: quantification pressure under meritocratic evaluation systems

At the macro-system level, psychological distress was embedded in broader cultural and policy logics that redefined academic worth in increasingly quantitative and competitive terms. Performance-oriented governance, meritocratic competition, and utilitarian academic culture did not simply intensify pressure in a general sense; they also reshaped the normative framework through which teachers understood worthwhile teaching and legitimate academic achievement. Within this macro-level environment, quantification pressure became educationally consequential because it encouraged teachers to interpret pedagogical work through institutional metrics rather than through developmental commitments to student learning. Psychological distress at this level should therefore be understood as a response to competition that is also tied to changing beliefs about what teaching is for and what counts as success in education.

Chinese higher education has increasingly adopted performance-oriented governance, closely intertwined with global ranking logics and national excellence initiatives such as the “Double First-Class” initiative. Within this policy environment, quantifiable indicators—especially SCI/SSCI publications and national-level grants—became dominant symbols of academic value. These arrangements intensified competition and redirected teachers’ attention and motivational resources away from pedagogical engagement toward externally regulated performance demands. Participants frequently described this landscape through the term *neijuan*, capturing a sense of escalating competition that undermined professional meaning. As one teacher observed, “Everyone is competing harder, but not necessarily producing better work. We’re just trying to survive the metrics” (P6). This macro-system pressure lies in the way it may reshape beliefs about teaching by positioning measurable outputs, rather than student development, as the most legitimate markers of academic success.

A salient institutional manifestation of this logic is the spread of meritocratic competition and the expansion of “up-or-out” systems. Although framed as reforms to enhance vitality and accountability, participants described local implementation as rigid and high-stakes, with the typical 3–5 years evaluation period experienced as an “all-or-nothing” wager: failure to meet narrowly defined thresholds could lead to contract termination. Such job insecurity is consistently associated with greater psychological distress, particularly in high-performance research environments (Shoss, 2017; Yang et al., 2024). Under these conditions, academic worth becomes tightly coupled with numerical output, fostering chronic anxiety and a sense of alienation.

These pressures were further complicated by cultural expectations rooted in Confucian traditions. Historically, the academic profession has been associated with the moral role of the *shi* (scholar-official), emphasizing ethical cultivation, social responsibility, and educational mentorship. Many participants reported entering academia with aspirations aligned with the ideal of “transmitting knowledge and cultivating virtue,” yet metric-driven evaluation practices produced pronounced identity tension. As one participant reflected, “I wanted to be a teacher who guides students, but now I feel like a worker on an academic assembly line” (P7). Moreover, in competitive systems where social recognition and status remain salient, falling behind peers was often experienced as shameful even when participants worked long hours, consistent with critiques that meritocratic ideologies can translate structural constraints into personalized feelings of inadequacy (Deem et al., 2007; Littler, 2018).

Macro-system ideologies also interacted with the utilitarian culture and commodification of academic labor, reorienting universities toward competitive enterprises where research is valued primarily for its exchange value and auditability (Mok, 2015). In this context, “good teaching” was widely perceived as secondary to measurable research outputs, and the absence of publications was interpreted as individual failure rather than a product of structural constraints. Participants described this realignment as producing a pervasive “loss of meaning,” as professional purpose became increasingly detached from education, mentorship, and intellectual exploration.

Meritocratic and market-oriented ideologies at the macro-system level cascade downward, shaping value conflicts in the exo-system, role confusion in the meso-system, and emotional exhaustion in the micro-system. Psychological distress among early career university teachers therefore emerges not merely as an individual response to heavy workload, but as a structural outcome of a higher education system increasingly organized around quantification, competition, and high-stakes evaluation. This macro-system quantification pressure may reshape teachers’ beliefs about worthwhile teaching and legitimate academic achievement. As academic worth becomes increasingly defined through output metrics, teaching may be reinterpreted as a secondary obligation rather than a developmental process of supporting learning, which may in turn influence how teachers prioritize, design, and evaluate instructional work.

### 4.5 Chrono-system: age anxiety shaped by cultural norms

At the chrono-system level, psychological distress was structured by temporal pressures that made early career life feel compressed, cumulative, and future-oriented. Age thresholds, probationary evaluation cycles, and culturally normative expectations surrounding career progression, marriage, and childbearing did not merely function as background conditions; they also shaped teachers’ sense of agency, persistence, and future-oriented motivation for teaching. In this sense, time operated not only as an amplifier of distress, but also as a force that reorganized how early career teachers interpreted professional possibility, allocated psychological energy, and sustained commitment to pedagogical development. Chrono-system pressures are therefore educationally significant because they may narrow the temporal space required for long-term instructional growth.

Participants emphasized the symbolic power of age in the academic labor market. Many research funding schemes and talent programs impose explicit age limits at 35, turning this threshold into a decisive deadline for academic legitimacy. As one participant remarked, “If I fail to secure a national-level grant before the age of 35, people assume that my academic future is over” (P3). This age-based benchmarking compressed early career trajectories into an accelerated timeline, pushing teachers toward rapid publication and grant acquisition immediately after graduation, often at the expense of health and wellbeing.

Consistent with this temporal compression, participants reported pronounced age-related stress reactions, including chronic insomnia, sudden weight changes, and acute anxiety episodes. These accounts resonate with evidence that perceived age discrimination and career time pressure are strongly associated with psychological distress and lower job satisfaction (Ng and Feldman, 2008). In China, the age-35 threshold has become a culturally embedded form of ageism, with those over 35 often viewed as less adaptable or less competitive and therefore facing structural barriers to mobility and advancement (Li and Tang, 2023). For early career university teachers, this discourse created a persistent sense of countdown, intensifying anxiety even during periods of apparent productivity.

Beyond chronological age, psychological distress also fluctuated across institutional evaluation cycles. Participants described a recurring emotional trajectory: early optimism and high achievement motivation, followed by frustration and self-doubt after repeated grant or manuscript rejections, and culminating in heightened vigilance and defensive coping as evaluation deadlines approached. Such patterns suggest that repeated experiences of limited control over evaluative outcomes can erode perceived agency and motivation over time, resembling dynamics described in work on learned helplessness (Seligman, 1975).

The months preceding formal evaluations were described as particularly exhausting, with anticipatory anxiety often expressed somatically through heart palpitations, sleep disturbances, and difficulty disengaging from work. One participant noted, “As the assessment gets closer, my body reacts before my mind—heart racing, high anxiety and sleep disturbance” (P1). Cyclical exposure to high-stakes assessment depleted emotional resources and undermined longer-term sustainability, consistent with research linking repeated performance monitoring to emotional strain (Maslach et al., 2001).

Chrono-system pressures were further compounded by culturally normative life-course expectations around marriage, childbearing, and caregiving. For women, these pressures intersected sharply with medical and social discourses surrounding “advanced maternal age,” commonly defined as childbirth at age 35 or above (Wang et al., 2022), and associated psychological stress risks (Li et al., 2024). Female participants described acute temporal conflicts as they attempted to synchronize academic productivity with socially sanctioned timelines for motherhood. More broadly, these experiences reflect the force of the “social clock”—normative expectations regarding when life events should occur (Neugarten et al., 1965). Ecologically, participants described age norms, evaluation timelines and culturally normative life-course expectations as interacting with the other system levels in ways that amplified their sense of distress over time. From an educational-psychology mechanism perspective, repeated rejection, age-based deadlines, and compressed evaluation cycles are consequential because, over time, they may undermine teachers’ sense of agency and self-efficacy, weaken their persistence, and reduce their future-oriented motivation for teaching (Bandura, 2001; Ryan and Deci, 2000). Participants’ accounts suggest that such conditions may encourage short-term, defensive, and performance-focused coping, leaving less psychological space for sustained pedagogical engagement and developmental investment in teaching. The chrono-system therefore acted as a temporal amplifier that intensified strains originating elsewhere in the ecology, allowing emotional exhaustion, role confusion, value conflict, and quantification pressure to accumulate across time.

### 4.6 Ecological psychological distress cascade: interlocking mechanisms across systemic layers

**FIGURE 1:**
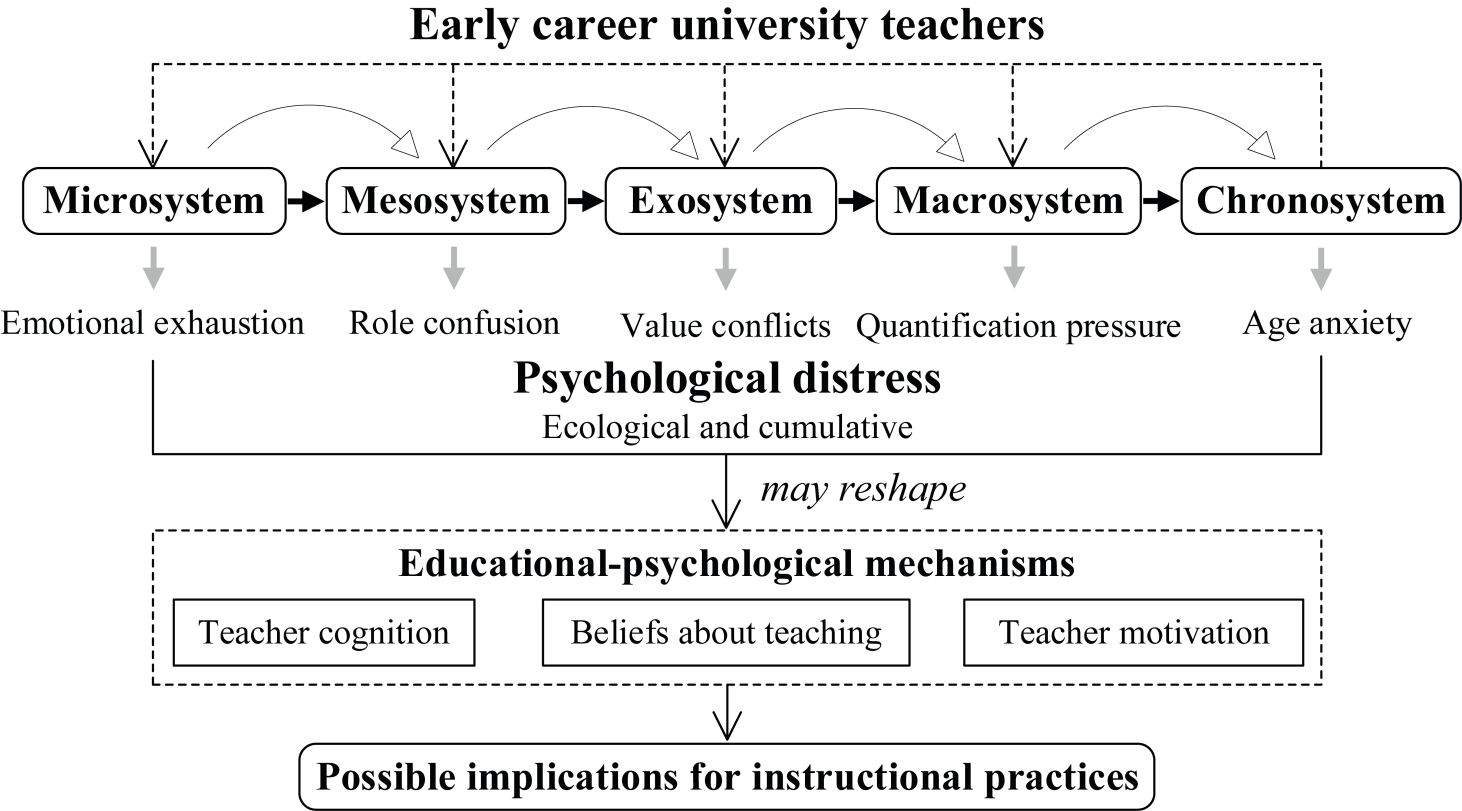
Ecological psychological distress cascade and its educational-psychological mechanisms.

Synthesizing findings across the micro-, meso-, exo-, macro-, and chrono-systems, this study interprets participants’ accounts through the lens of an ecological cascade: a multi-layered pattern in which pressures across systemic levels were described as interrelated and mutually reinforcing. Psychological distress therefore does not stem from isolated stressors or individual vulnerability; rather, it is produced through interlocking mechanisms involving emotional exhaustion, role confusion, value conflicts, quantification pressures, and age anxiety. The findings do not suggest a direct or immediate causal link between distress and teaching outcomes. Instead, they show how distress gradually alters the psychological conditions under which teaching is planned, interpreted, and enacted. Three interrelated educational-psychological mechanisms are especially important here: teacher cognition, teacher motivation, and beliefs about teaching. As illustrated in Figure 1, the ecological systems identified in this study provide the contextual conditions under which distress emerges, while these educational-psychological mechanisms help explain how distress may influence teaching practices.

At the micro-system level, psychological distress is first encountered in emotionally demanding daily practices, particularly interactions with students, collegial relationships, and leadership expectations. Participants described investing substantial affective, cognitive, and temporal labor to meet immediate expectations while receiving limited recognition and few opportunities for recovery. These proximal strains gradually gave rise to emotional exhaustion over time. They then spilled into the meso-system, where multiple roles—teaching, research, administrative service, and family responsibilities—collided. Misalignment across these domains transformed momentary stress into chronic role conflict, as finite personal time had to meet simultaneous demands for high research output, competent teaching, and institutional responsiveness. In ecological terms, meso-system tensions amplified micro-system exhaustion rather than merely adding another burden (Bronfenbrenner, 1979).

The exo-system magnified the cascade by institutionalizing managerialist governance. Administrative expansion, uneven task distribution, and research resource allocation reorganized academic labor into a continuously audited activity, intensifying documentation, procedural compliance, and metric visibility. For early career university teachers with limited decision-making power, these arrangements fostered depersonalization and reduced perceived control over professional life. As managerialism translated policy rationalities into everyday practices (Clarke and Newman, 1997; Deem, 1998), psychological distress became normalized and internalized. Participants reported learning to self-discipline, suppress frustration, and prioritize compliance over professional judgment, patterns consistent with critiques of neoliberal governance in higher education (Ball, 2012; Giroux, 2014).

At the macro-system level, meritocratic competition and utilitarian culture provided the normative justification for these arrangements. Quantified indicators—publication counts, journal rankings, grant income—became moralized measures of worth, framing success and failure as personal responsibility. This ideological framing obscured structural inequality while intensifying competition, captured in participants’ frequent use of *neijuan*. Consistent with critiques of meritocracy, such systems heightened distress by reinterpreting systemic constraints as individual inadequacy (Littler, 2018). In this sense, macro-system ideology legitimized exo-system governance, normalized meso-system overload, and rendered micro-system strain psychologically meaningful as a test of personal competence.

The chrono-system highlights the temporal dimension through which participants experienced these pressures as cumulative and anticipatory. Age thresholds, probationary evaluation cycles, and culturally normative life-course timelines compressed early career trajectories and heightened perceived stakes. Participants’ emotions were directed toward present tasks and toward uncertain futures shaped by rigid deadlines, including promotion windows, funding eligibility cut-offs, and “up-or-out” evaluation periods. This temporal compression cultivated persistent vigilance and anxiety, and repeated rejection or delayed success could erode perceived control in ways reminiscent of learned helplessness dynamics (Seligman, 1975). Chrono-system pressures accelerated the cascade by limiting opportunities for recovery and recalibration, and by narrowing the space for developmental pacing or alternative career imaginaries.

Across systems, the cascade operated through resource depletion and negative feedback loops. Emotional exhaustion weakened coping capacity; role confusion eroded restorative boundaries; managerialist governance cultures diminished autonomy; meritocratic ideology intensified self-blame; and temporal norms foreclosed patience and experimentation. This pattern aligns with conservation of resources theory, which argues that sustained resource loss increases vulnerability to stress and burnout (Demerouti, 2025). The distinctive contribution of the present findings is to show that such resource depletion unfolds ecologically—across relational, institutional, ideological, and temporal domains—rather than within the individual alone. Crucially, the cascade culminated in anticipatory psychological distress, oriented toward imagined futures of evaluation failure, stalled advancement, or forced exit from academia. This extends classic perspectives focused on present exhaustion and depersonalization (Maslach and Jackson, 1981) by demonstrating how institutional and cultural “clocks” organize emotional experience and sustain distress over time.

The cascade identified here should not be read as a simple causal chain. Rather, it describes how different pressures accumulate and gradually reshape the psychological conditions under which teaching takes place. At the micro-system level, strained interpersonal experiences may alter how teachers perceive and interpret classroom demands, weaken instructional self-efficacy, and increase defensive emotion regulation, thereby reducing confidence in classroom intervention and pedagogical responsiveness. At the meso-system level, sustained role conflict across teaching, research, administrative work, and family life may fragment teachers’ attentional resources and weaken their motivation for pedagogical engagement, leaving less time, energy, and commitment for lesson planning and student support. At the exo-system level, managerialist evaluation practices and unequal resource allocation may reduce teachers’ perceived autonomy and weaken the intrinsic motivation needed for sustained investment in teaching, as pedagogical work is often symbolically valued but practically marginalized. At the macro-system level, quantification pressure and meritocratic competition may reshape teachers’ beliefs about “good teaching” and “legitimate academic achievement,” encouraging them to prioritize measurable research outputs over developmental commitments to student learning. At the chrono-system level, repeated rejection, age-based deadlines, and compressed evaluation cycles may gradually erode teachers’ sense of agency, persistence, and future-oriented motivation for teaching, pushing them toward short-term, defensive, and performance-focused coping. These psychological changes may, in turn, encourage more defensive, compliance-oriented, or less interactive instructional practices. The ecological cascade therefore captures how distress accumulates and how relational, institutional, cultural, and temporal pressures become consequential for everyday teaching.

These findings also invite comparison with research from other higher education contexts. Prior studies in Western academia have likewise linked early career academics’ distress to managerial governance, workload intensification, precarious employment, and work–family tensions (Loveday, 2018; Nicholls et al., 2022). By contrast, the present study suggests that, in mainland China, these pressures are further compounded by the interaction of national excellence policies, performance-oriented reforms, age-related career thresholds, and culturally embedded expectations surrounding academic success (Zhang et al., 2024; Lin, 2025). Such comparison indicates that psychological distress among early career university teachers is shaped by both shared global pressures and context-specific ecological configurations. Accordingly, addressing psychological distress among early career university teachers requires interventions that disrupt the cascade at multiple points—rebalancing evaluation regimes, recognizing invisible labor, restoring professional autonomy, and increasing temporal flexibility—rather than relying primarily on individual coping strategies.

## 5 Conclusion

This study examined psychological distress among early career university teachers in mainland China through an ecological lens. Rather than treating psychological distress as an individual deficit or a collection of isolated stressors, the study reframes it as an ecological, cumulative, and future-oriented process shaped by the interplay of relational, institutional, sociocultural, and temporal conditions (Bronfenbrenner, 1979). In this sense, psychological distress emerged not simply from excessive workload, but from the way multiple pressures converged, accumulated, and became increasingly difficult to manage over time.

At a theoretical level, this study extends the application of Bronfenbrenner’s ecological systems theory to the study of teacher psychological distress in higher education. By conceptualizing distress as an ecological and cumulative process, the study moves beyond individual-centered or single-variable explanations and reveals how distress is produced through interlocking pressures across systemic layers. The notion of an ecological cascade helps explain how emotional exhaustion, role confusion, value conflict, quantification pressure, and age anxiety become mutually reinforcing across relational, organizational, ideological, and temporal levels. In particular, the findings highlight the chrono-system not as a passive background dimension, but as a temporal amplifier that reorganizes distress into an anticipatory and future-oriented form. This temporal emphasis adds a dynamic dimension to existing ecological understandings of teacher wellbeing and resonates with research on resource depletion, burnout, and time-related institutional and cultural pressures (Maslach et al., 2001; Demerouti, 2025).

At an empirical level, the study offers a context-sensitive account of early career university teachers’ psychological distress in mainland China. It shows that such distress is shaped within a distinctive institutional ecology marked by performance-oriented governance, “Double First-Class” reforms, “up-or-out” evaluation systems, metricized definitions of academic worth, and culturally salient age-related expectations (Zheng and Li, 2025; Lin, 2025). Although some of these pressures resonate with findings from other higher education contexts, the present study suggests that, in mainland China, their interaction produces a particularly compressed and competitive early career trajectory. Importantly, the study also clarifies why psychological distress should be considered not only a teacher wellbeing issue but also a condition with possible implications for instructional practices. By linking this institutional and cultural configuration to teacher cognition, teacher motivation, and beliefs about teaching, the study contributes to educational psychology by showing how contextual pressures in higher education may become consequential for the psychological conditions under which teaching is planned, interpreted, and enacted.

By foregrounding psychological distress as an ecological phenomenon, this study reframes early career university teachers’ psychological distress as a systemic challenge requiring coordinated interventions beyond individual coping. Practically, the findings point to the need for institutional conditions that protect teachers’ capacity to sustain pedagogical engagement and instructional responsiveness, rather than treating psychological distress solely as an individual wellbeing issue. The findings suggest that universities need to revise rigid performance-oriented arrangements and “up-or-out” thresholds, while also building more supportive institutional environments through clearer workload allocation, more transparent access to research resources, and fairer recognition of teaching, mentoring, and administrative labor. In addition, universities should strengthen local support infrastructures through formal mentoring schemes, peer-support communities, and psychologically safe departmental climates that can buffer emotional exhaustion and relational isolation. Given the findings related to the chrono-system, universities should also offer more humane pacing of probationary expectations, greater temporal flexibility, and career-planning support tailored to different stages of early career teachers’ professional development. Such institutional support mechanisms are valuable because they can interrupt the ecological cascade through which psychological distress accumulates over time.

Several limitations should be acknowledged. First, the study is based on a small qualitative sample of 12 early career university teachers in Sichuan Province, so the findings are analytically rather than statistically generalizable. Second, because the data rely on retrospective interviews, the study cannot fully capture how distress develops across longer career trajectories and policy cycles. Third, the institutional and cultural specificity of mainland China means that some findings may not transfer directly to other higher education systems. Future research could therefore adopt longitudinal and cross-context comparative designs to examine how ecological pressures on early career university teachers evolve across time and across institutional settings. Overall, psychological distress among early career university teachers should be understood not as a matter of individual resilience alone, but as a systemic condition of contemporary teaching.

## Author’s note

1. “Double First-Class” (双一流) refers to a national higher education policy initiative launched by the Chinese government to promote the development of world-class universities and world-class disciplines. Replacing the earlier Project 211 and Project 985 schemes, it has intensified performance-based governance, resource concentration, and inter-university competition in Chinese higher education.

2. “Up-or-out” (非升即走) refers to a probationary evaluation regime in which early career university teachers are required to meet specified performance criteria within a fixed period in order to retain their positions; failure to do so may result in contract non-renewal or termination. In the context of Chinese higher education, this regime is closely associated with intensified publication pressure, job insecurity, and accelerated career timelines.

## Data Availability

The original contributions presented in this study are included in the article/supplementary material, further inquiries can be directed to the corresponding author.

## Appendix

In this interview, when we refer to “psychological distress,” we mean experiences such as feeling stressed, anxious, frustrated, depressed, or emotionally strained in a way that affects your day-to-day functioning. This does not necessarily imply a clinical diagnosis. Please feel free to describe your experiences in your own words, using whatever terms feel most natural to you.

**APPENDIX TABLE 1:** Experiences of psychological distress among early career university teachers (*N* = 12).

Ecological system	Interview questions
Background information	1. Could you briefly describe your work over the past year? 2. Over the past 6 months, what kinds of experiences have felt most challenging or emotionally taxing for you?
Micro-system	In your day-to-day interactions with students, colleagues, and leaders, which situations tend to be the most stressful or demanding? Could you describe a recent specific incident in detail?
Meso-system	1. Have you experienced difficulties in balancing teaching and research responsibilities? If yes, could you describe typical scenarios and how you cope with them? 2. How would you describe the boundary between your work and personal life? In what scenarios is this boundary most likely to be challenged or blurred?
Exo-system	1. In your view, what constitutes a “good” university teacher? Does the current evaluation system align with your own understanding? If not, in what ways does it differ? 2. Regarding access to resources (e.g., research projects, funding, platforms/labs, administrative support), how do these processes typically operate from your perspective?
Macro-system	1. How do performance-oriented evaluation and promotion processes work in your institution from your perspective? 2. What expectations do people around you (e.g., colleagues, family members, society) have of you? How do these expectations shape your decisions and emotions?
Chrono-system	1. Do you feel any pressure related to age (e.g., age thresholds or time-limited opportunities)? How does this pressure manifest in your day-to-day decisions? 2. How does your distress level or emotional experience change over the course of the academic year?
